# Bilateral Simultaneous Chronic Achilles Tendon Rupture Treated With Open and Endoscopic Flexor Hallucis Longus Tendon Transfer: A Case Report

**DOI:** 10.7759/cureus.106936

**Published:** 2026-04-13

**Authors:** Abdelrahman A Afifi, Mahmoud A Gamal, Awab A Elaslaby

**Affiliations:** 1 Department of Orthopaedic Surgery, Faculty of Medicine, Ain Shams University, Cairo, EGY

**Keywords:** achilles tendon rupture, bilateral achilles rupture, chronic achilles rupture, endoscopic achilles reconstruction, flexor hallucis longus transfer, foot and ankle surgery, open achilles reconstruction, tendon transfer

## Abstract

Bilateral chronic Achilles tendon rupture is an exceptionally rare condition with limited evidence guiding optimal management. We present a unique within-patient comparison of two reconstructive techniques: endoscopic flexor hallucis longus (FHL) tendon transfer on one side and open FHL transfer on the contralateral side. This design eliminates interpatient variability and allows direct comparison of operative time, surgical exposure, cosmetic outcomes, and functional recovery. Functional outcomes were assessed at 12 months and confirmed at the 24-month follow-up. Both techniques resulted in excellent restoration of plantar flexion strength and high patient satisfaction. The endoscopic approach required longer operative time but appeared to offer better cosmetic outcomes based on wound appearance. This case suggests that both open and endoscopic FHL tendon transfer may be effective options for chronic Achilles tendon rupture; however, further studies are needed to validate these findings.

## Introduction

Chronic Achilles tendon rupture is a challenging clinical condition associated with significant functional impairment, including weakness in plantarflexion, reduced push-off strength, and altered gait mechanics [1]. It is commonly defined as a rupture presenting more than four to six weeks after the initial injury, at which stage tendon retraction, poor tissue quality, and defect formation often preclude primary end-to-end repair and necessitate reconstructive procedures [1,2].

Flexor hallucis longus (FHL) tendon transfer is a well-established reconstructive option for chronic Achilles tendon rupture due to its anatomical proximity, similar line of pull, and ability to restore dynamic plantarflexion during gait [3,4]. This technique is particularly indicated in cases with large tendon defects, where direct repair is not feasible.

Both open and minimally invasive approaches have been described for FHL tendon transfer. The open technique allows direct visualization and secure fixation, while endoscopic and minimally invasive techniques aim to reduce soft tissue disruption and wound-related complications [5]. However, these techniques are technically demanding and require specialized expertise.

Bilateral chronic Achilles tendon rupture is an exceptionally rare clinical event, with limited data available to guide optimal management or compare surgical techniques within the same patient. In this report, we present such a rare case of bilateral chronic Achilles tendon rupture treated using both open and endoscopic FHL tendon transfer techniques in a single operative session, allowing a direct within-patient comparison of clinical and functional outcomes.

## Case presentation

A 57-year-old female patient presented to our clinic three months after sustaining a fall following a minor domestic injury. Initial evaluation at an outside facility shortly after the injury, including magnetic resonance imaging (MRI), confirmed bilateral Achilles tendon ruptures, and surgical intervention was recommended at that time. However, the patient deferred treatment for approximately three months due to occupational commitments. Definitive surgical management was performed one week after presentation to our institution. Her medical history was significant for well-controlled hypertension. There was no history of corticosteroid use, fluoroquinolone exposure, inflammatory arthropathy, or prior Achilles tendon pathology.

On clinical examination, bilateral calf muscle wasting was evident, with reduced active plantarflexion strength. The Thompson test was positive on both sides, consistent with Achilles tendon rupture. The patient was unable to perform both single-leg and bilateral heel rise tests. Gait assessment demonstrated reduced push-off strength and functional limitation (Videos 1-3).

**Video 1:** Preoperative Gait Assessment Preoperative gait demonstrating bilateral weakness and reduced push-off strength, consistent with chronic Achilles tendon rupture.

**Video 2:** Preoperative Thompson Test (Bilateral) Bilateral Thompson test demonstrating absence of plantarflexion, consistent with complete Achilles tendon rupture on both sides.

**Video 3:** Preoperative Bilateral Heel Rise Test Patient unable to perform bilateral heel rise, indicating severe functional impairment due to chronic Achilles tendon rupture.

Functional outcomes were assessed using validated scoring systems, including the American Orthopaedic Foot and Ankle Society (AOFAS) hindfoot score and the Foot and Ankle Ability Measure (FAAM) [6,7]. Preoperative evaluation demonstrated marked functional impairment, with an AOFAS score of 43 and a FAAM score of 54.4%. A summary of clinical and functional outcomes across preoperative, 12-month, and 24-month follow-up time points is presented in Table 1. Outcome measures were recorded at the patient level, and side-specific assessment was not performed. This represents a limitation of data collection rather than an inherent limitation of the scoring systems.

**Table 1:** Summary of Clinical and Functional Outcomes at Preoperative, 12-Month, and 24-Month Follow-up Time Points AOFAS: American Orthopaedic Foot and Ankle Society; FAAM: Foot and Ankle Ability Measure; VAS: Visual Analog Scale; MRC: Medical Research Council. Outcome measures were recorded at the patient level and were not assessed separately for each side

Parameter	Preoperative	12 Months Follow-up	24 Months Follow-up
AOFAS Hindfoot Score	43	95	100
FAAM Score (%)	54.40%	97.70%	99.70%
VAS Pain Score	N/A	3/10 (at 3 months)	0–1/10
Plantarflexion Strength (MRC)	Weak	5/5	5/5
Heel Rise Test	Unable	Achieved	Maintained
Gait	Impaired	Improved	Normal
Thompson Test	Positive	Negative	Negative
Wound Complications	N/A	None	None

**Figure 1:**
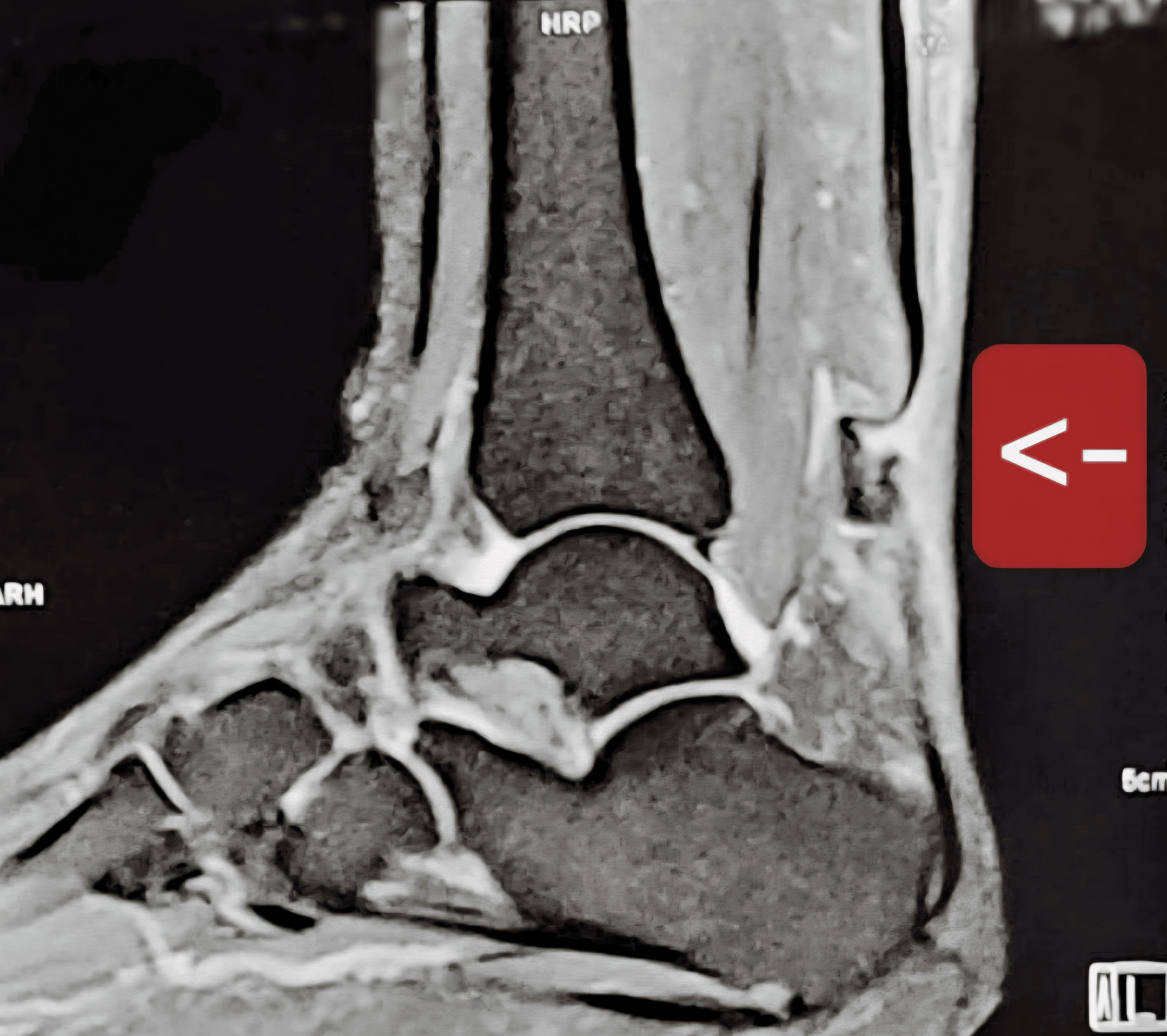
Title: Right Ankle MRI (Sagittal View) Demonstrating Chronic Achilles Tendon Rupture Sagittal MRI of the right ankle demonstrating complete discontinuity of the Achilles tendon (arrow) with proximal tendon retraction and interposed degenerative tissue, consistent with a chronic rupture. The tendon defect (gap size) is estimated to exceed 5 cm, supporting the need for reconstructive management rather than primary repair.

**Figure 2:**
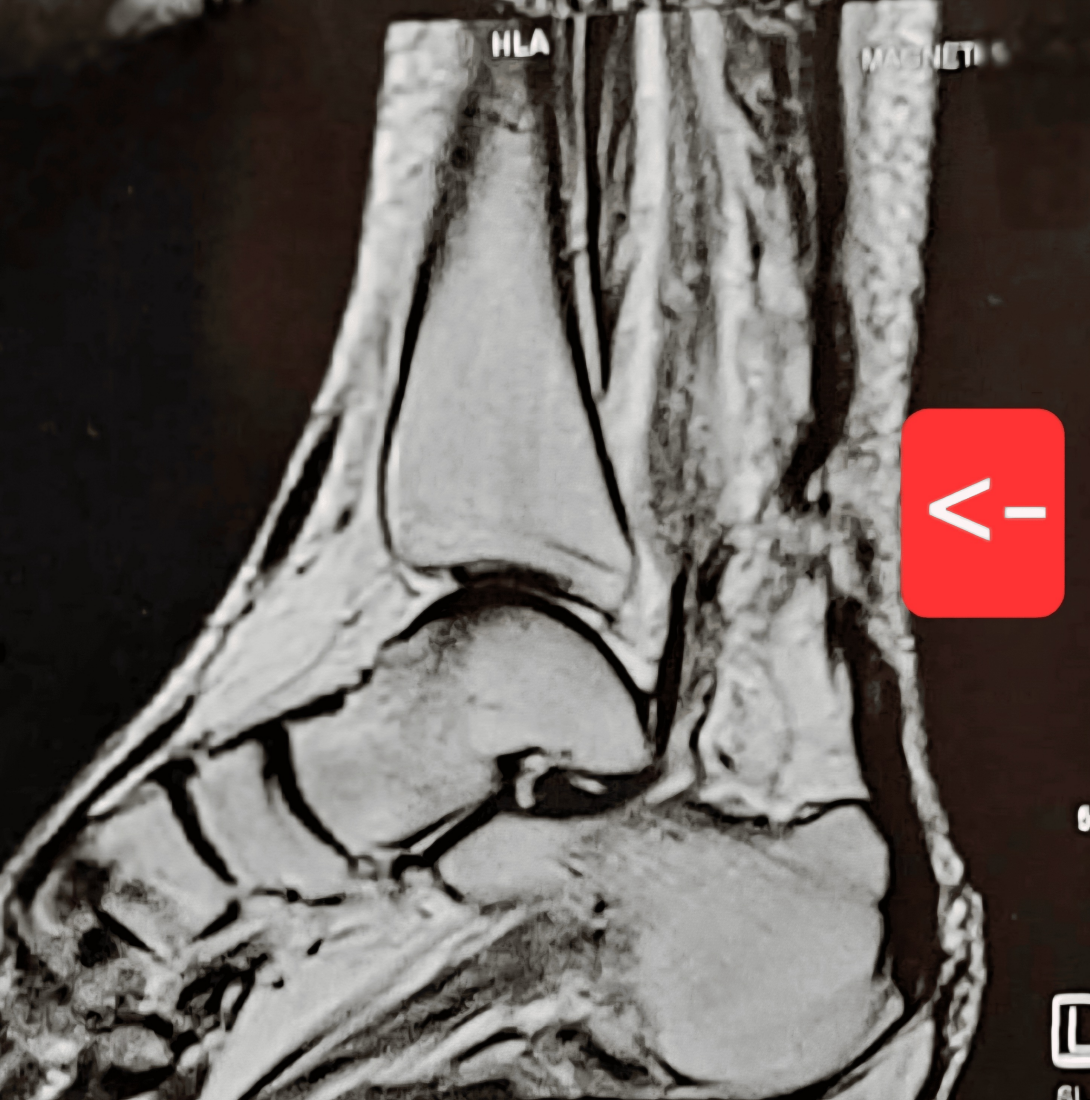
Left Ankle MRI (Sagittal View) Demonstrating Chronic Achilles Tendon Rupture Sagittal MRI of the left ankle demonstrating complete discontinuity of the Achilles tendon (arrow) with proximal tendon retraction and interposed degenerative tissue, consistent with a chronic rupture. The tendon defect (gap size) is estimated to exceed 5 cm.

Magnetic resonance imaging (MRI) confirmed chronic bilateral Achilles tendon ruptures, demonstrating complete tendon discontinuity with proximal retraction and interposed degenerative tissue on both sides (Figures 1, 2). The tendon defect (gap size) was estimated to exceed 5 cm bilaterally, consistent with a chronic rupture and explaining the unsuitability of primary end-to-end repair.

Given the chronic nature of the injury, poor tendon quality, and large defect size, surgical reconstruction was indicated. After a detailed discussion of treatment options, risks, and expected outcomes, a decision was made to proceed with single-stage bilateral reconstruction using two different surgical techniques. This approach aimed to reduce overall rehabilitation time and allow a direct within-patient comparison while minimizing interpatient variability. Bilateral reconstruction was performed during the same operative session. Intraoperative findings confirmed a tendon defect exceeding 5 cm on both sides.

Endoscopic FHL tendon transfer was performed on the right side. Posterior ankle endoscopy was carried out using standard posteromedial and posterolateral portals. Fibrotic tissue at the rupture site was debrided, and the FHL tendon was identified, released distally, and harvested under endoscopic visualization. A calcaneal bone tunnel was created at the anatomical Achilles insertion, and the tendon was transferred and fixed using an interference screw with the ankle held in slight plantarflexion. The operative time was approximately 60-65 minutes.

Open FHL tendon transfer was performed on the left side through a posteromedial longitudinal incision. Degenerated tendon tissue was excised, and the FHL tendon was harvested under direct visualization. A calcaneal bone tunnel was prepared, and the tendon was transferred and secured using an interference screw. Adequate tension and restoration of resting plantarflexion were confirmed prior to wound closure. The operative time was approximately 25-30 minutes.

**Figure 3:**
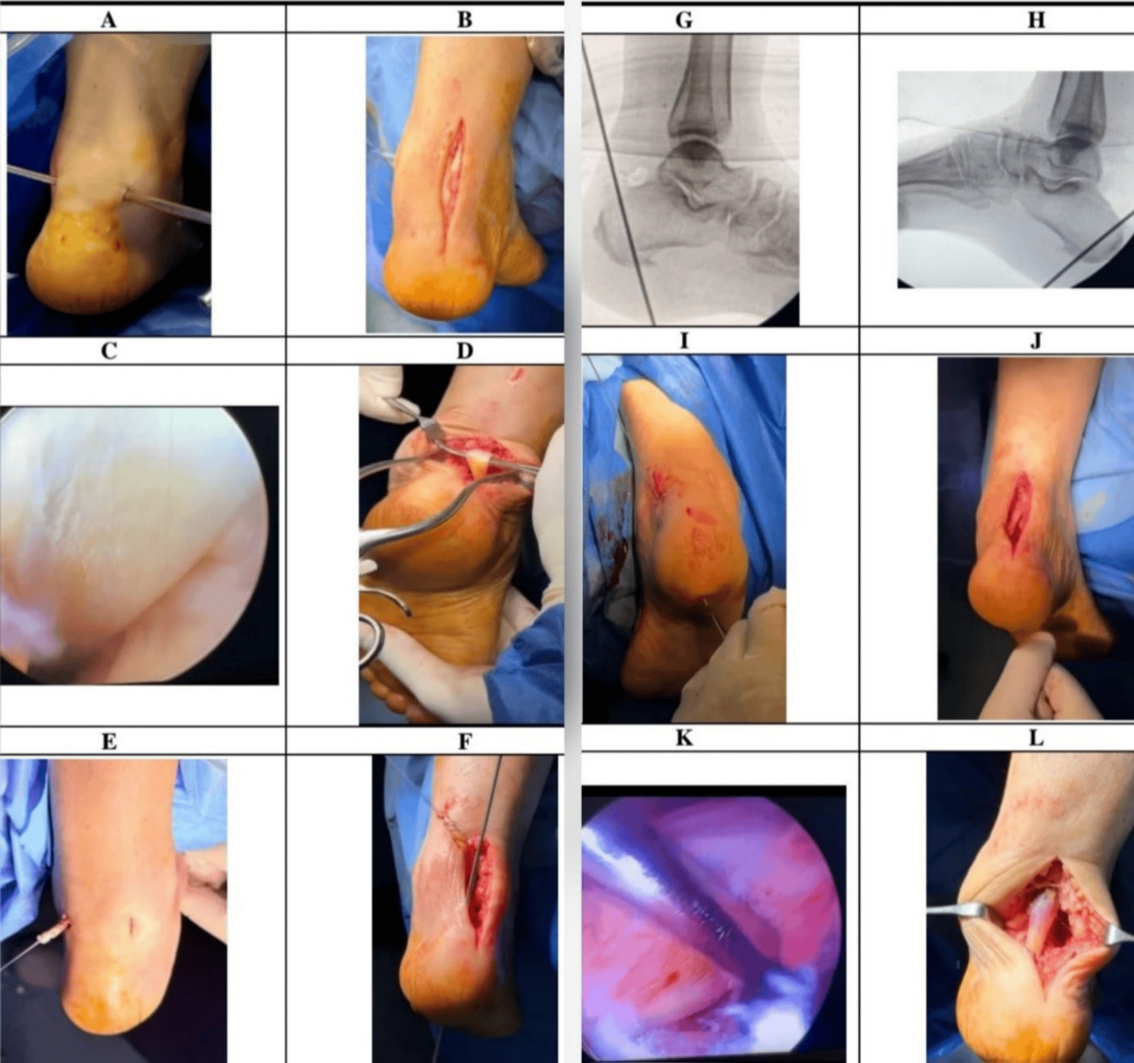
Intraoperative Comparison of Endoscopic and Open Achilles Reconstruction Using Flexor Hallucis Longus Transfer Intraoperative photographs demonstrating corresponding operative steps of flexor hallucis longus (FHL) tendon transfer using endoscopic and open techniques. Panels A, C, E, G, I, and K illustrate sequential steps of the endoscopic technique: (A) Establishment of posteromedial and posterolateral portals, (C) Identification of the FHL tendon within the posterior ankle compartment, (E) Suture control and mobilization of the tendon, (G) Intraoperative fluoroscopic confirmation of calcaneal bone tunnel position, (I) Transfer of the tendon into the calcaneal bone tunnel with endoscopic confirmation of appropriate positioning, (K) Fixation using an interference screw and final endoscopic assessment. Panels B, D, F, H, J, and L demonstrate the corresponding steps performed using the open technique: (B) Exposure and identification of the FHL tendon through a posteromedial approach, (D) Tendon harvest at the level of the sustentaculum tali, (F) Preparation of the calcaneal bone tunnel, (H) Intraoperative fluoroscopic confirmation of bone tunnel position, (J) Tendon transfer and fixation within the calcaneal tunnel using an interference screw, (L) Final confirmation of FHL tendon position within the calcaneal tunnel. The panels are arranged in a grid format, allowing direct visual comparison between corresponding steps of the endoscopic and open techniques.

The difference in operative time may have been influenced by procedural sequence, as the endoscopic procedure was performed first and required additional setup. A comparison of intraoperative and technical characteristics between the two techniques is shown in Figure 3 and summarized in Table 2.

**Table 2:** Intraoperative Comparison Between Endoscopic and Open Techniques Comparison reflects intraoperative and observational findings within a single patient. Direct statistical comparison is limited due to the case report design.

Parameter	Endoscopic (Right)	Open (Left)
Operative Time	60–65 minutes	25–30 minutes
Surgical Approach	Endoscopic	Open
Visualization	Endoscopic	Direct
Fixation Method	Interference screw	Interference screw
Wound Size	Smaller	Larger
Wound Complications	None	None
Procedure Sequence	First	Second

**Figure 4:**
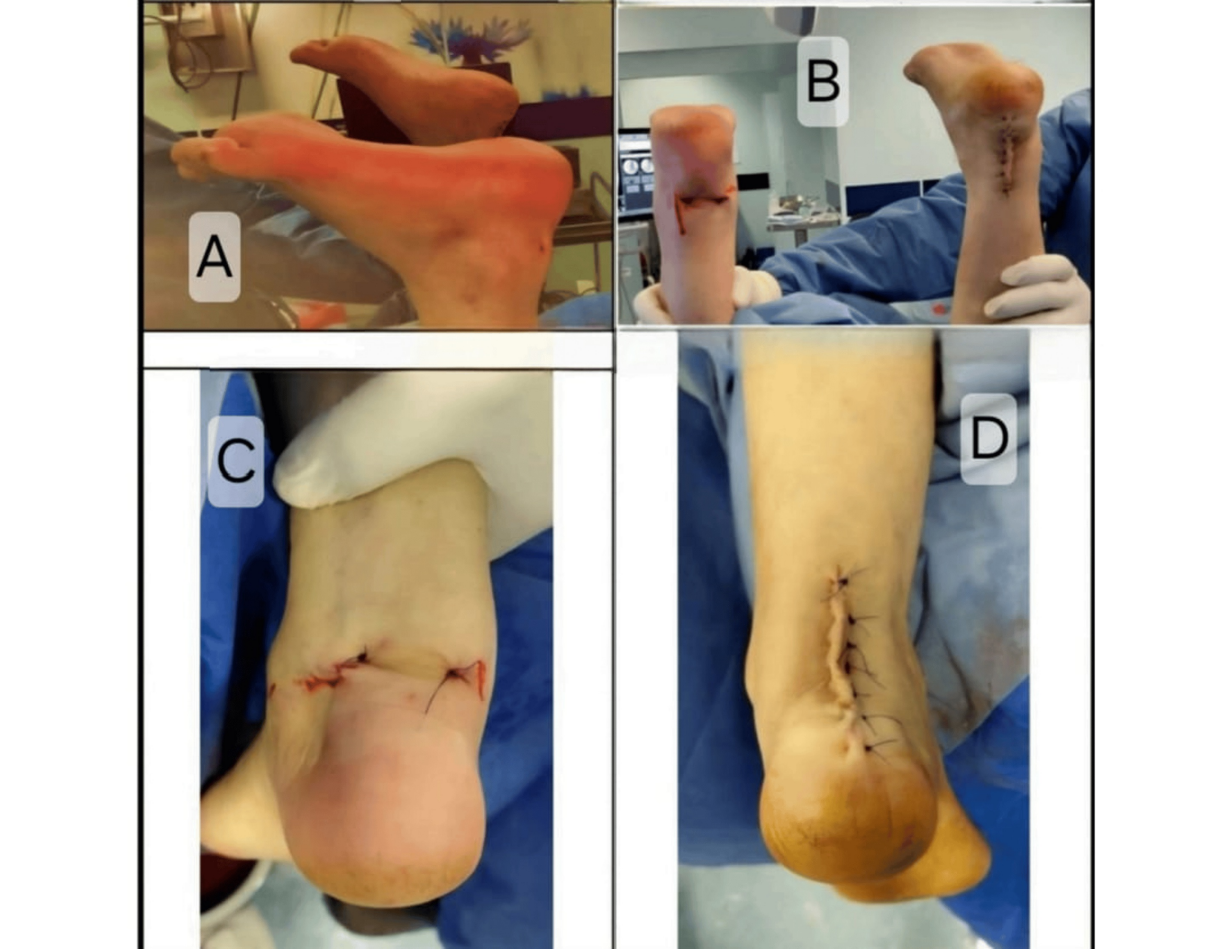
Post-transfer Alignment and Wound Appearance Following Achilles Reconstruction Assessment of resting ankle plantarflexion and final wound appearance following flexor hallucis longus tendon transfer. Panels A and B demonstrate postoperative plantarflexion alignment comparing endoscopic and open techniques. Panels C and D show final skin appearance following wound closure for the endoscopic and open approaches, respectively.

Both ankles were immobilized in plantarflexion immediately postoperatively. The postoperative course was uneventful, with no wound complications observed. A standardized rehabilitation protocol was followed. The patient was immobilized for two weeks, followed by gradual range-of-motion exercises. Partial weight-bearing was initiated at four weeks, progressing to full weight-bearing by six to eight weeks. Strengthening exercises were introduced at approximately 8-10 weeks postoperatively. Post-transfer alignment and wound appearance following Achilles reconstruction are shown in Figure 4.

At the three-month follow-up, the patient reported a visual analog scale (VAS) pain score of 3/10. This value reflects overall patient-reported pain and was not assessed separately for each side, which represents a limitation of the reported pain data. The patient demonstrated progressive functional improvement and returned to work at 16 weeks postoperatively.

At the 12-month follow-up, clinical examination demonstrated restoration of plantarflexion strength (Medical Research Council grade 5/5) bilaterally. The patient was able to perform both bilateral and single-leg heel rise tests. Functional outcomes improved significantly, with an AOFAS score of 95 and an FAAM score of 97.7%.

At the 24-month follow-up, sustained recovery was observed, with full plantarflexion strength bilaterally (MRC 5/5) and no late complications. Functional scores reached an AOFAS score of 100 and an FAAM score of 99.7%. Functional recovery was further demonstrated by restoration of normal gait, successful heel rise, and a negative Thompson test bilaterally (Videos 4-9). These outcomes are summarized in Table 1. No clinically significant hallux weakness or functional limitation was observed during follow-up; however, formal quantitative assessment of donor site morbidity was not performed.

**Video 4:** Postoperative Gait Assessment at Two-Year Follow-up Gait assessment at two-year follow-up demonstrating symmetrical walking pattern with restored push-off strength.

**Video 5:** Postoperative Bilateral Heel Rise Test at Two-Year Follow-up Successful bilateral heel rise demonstrating restoration of functional plantarflexion strength.

**Video 6:** Postoperative Thompson Test (Right Side) Right-sided Thompson test demonstrating restoration of plantarflexion following Achilles reconstruction.

**Video 7:** Postoperative Thompson Test (Left Side) Left-sided Thompson test demonstrating restoration of plantarflexion

**Video 8:** Postoperative Plantarflexion Strength (Right Ankle) Clinical assessment demonstrating grade 5 plantarflexion strength of the right ankle at two-year follow-up.

**Video 9:** Postoperative Plantarflexion Strength (Left Ankle) Clinical assessment demonstrating grade 5 plantarflexion strength of the left ankle at two-year follow-up.

## Discussion

The present case provides a unique within-patient comparison of open and endoscopic FHL tendon transfer techniques for the management of bilateral chronic Achilles tendon rupture. By performing both techniques in the same patient during a single operative session, several confounding variables, including age, biological healing potential, comorbidities, and rehabilitation compliance, were partially controlled. However, factors such as tendon gap size, degree of degeneration, limb dominance, and intraoperative sequence may still have influenced outcomes. Nevertheless, this design allows for a more direct comparison than conventional inter-patient studies.

Both techniques resulted in excellent clinical and functional outcomes. Restoration of plantarflexion strength, marked improvement in AOFAS and FAAM scores, and high patient satisfaction were achieved at both short- and long-term follow-ups. Although the endoscopic technique required a longer operative time, it appeared to offer improved cosmetic outcomes and had the theoretical advantage of reduced soft tissue disruption. In contrast, the open technique remains technically straightforward, time-efficient, and allows direct visualization and secure fixation.

Traditional open techniques are associated with wound complications, particularly in patients with compromised soft tissue envelopes [8]. In the present case, no wound complications were observed, likely reflecting the patient’s relatively preserved soft tissue condition and absence of major risk factors. In contrast, endoscopic and minimally invasive techniques have been increasingly utilized to reduce surgical morbidity and soft tissue disruption [9]. However, these techniques are technically demanding and require a learning curve.

A standardized rehabilitation protocol was followed in accordance with established principles after Achilles tendon reconstruction [10,11], which likely contributed to the excellent functional recovery observed in this case.

This case also highlights the clinical challenges associated with delayed presentation of Achilles tendon rupture, including tendon retraction and compromised tissue quality, often necessitating reconstructive procedures rather than primary repair. These findings underscore the importance of early recognition and timely intervention to optimize outcomes.

The primary limitation of this report is its nature as a single case, which limits generalizability. Additionally, outcomes were recorded at the patient level and were not assessed separately for each ankle, limiting direct side-specific comparison between techniques. The allocation of surgical technique to each side was not randomized, and operative time may have been influenced by procedural sequence. Donor site morbidity was not formally evaluated, and outcome assessment was not blinded. Despite these limitations, this case provides valuable insight through a direct within-patient comparison of two established surgical techniques.

Clinical takeaway

Both open and endoscopic FHL tendon transfer techniques are effective options for managing chronic Achilles tendon rupture. While the endoscopic approach may reduce soft tissue morbidity, the open technique remains reliable and time-efficient. The choice of technique should be individualized based on surgeon's expertise and patient-specific factors [12].

## Conclusions

Bilateral chronic Achilles tendon rupture is a rare and challenging condition that requires careful clinical assessment and appropriate surgical planning. This case demonstrates that both open and endoscopic FHL tendon transfer techniques can achieve excellent functional outcomes, even in delayed presentations. The within-patient comparison suggests that while the endoscopic approach may offer potential advantages in soft tissue handling, this observation is based on qualitative assessment rather than objective outcome measures. The open technique remains reliable, time-efficient, and allows direct visualization and secure fixation.

No clinically significant donor site morbidity was observed; however, formal quantitative assessment was not performed, which represents a limitation. Ultimately, the choice of surgical technique should be individualized based on the surgeon's expertise, available resources, and patient-specific factors. Early recognition and timely intervention remain essential to optimize outcomes and reduce the need for complex reconstructive procedures.
